# Seasonal and nutritional changes in the short form of the leptin receptor expression and VEGF system in the choroid plexus, arcuate nucleus, and anterior pituitary in MTS-leptin and resistin-treated sheep

**DOI:** 10.3389/fnins.2023.1291997

**Published:** 2023-11-28

**Authors:** Dorota Anna Zieba, Weronika Biernat, Malgorzata Szczesna, Katarzyna Kirsz, Tomasz Misztal

**Affiliations:** ^1^Laboratory of Biotechnology and Genomics, Department of Nutrition, Animal Biotechnology, and Fisheries, Agricultural University of Krakow, Kraków, Poland; ^2^Department of Animal Physiology, The Kielanowski Institute of Animal Physiology and Nutrition, Polish Academy of Sciences, Jabłonna, Poland

**Keywords:** MTS-leptin, resistin, choroid plexus, leptin receptor, nutrition, photoperiod, VEGF, sheep

## Abstract

The short form of the leptin receptor (LeptRa) plays a key role in the transport of leptin to the central nervous system (CNS). Here, MTS-leptin and recombinant ovine (ro) leptin-mediated expression of LeptRa and VEGFA and VEGFR2 concentration in selected hypothalamic nuclei, choroid plexus (ChP), and anterior pituitary (AP) were analyzed considering the photoperiod and acute-fasting (experiment 1), and nutritional status (experiment 2) of ewes. In experiment 1, 60 sheep were fed normally or fasted for 72 h and received one injection of saline, MTS-leptin, or roleptin 1 h prior to euthanasia. LeptRa mRNA transcript levels and VEGF system protein concentrations were detected in the ARC, ChP predominantly in the SD, and AP for the LD without detection of LeptRa in the POA and VMH/DMH. In experiment 2, an altered diet for 5 months created lean or fat sheep. Twenty sheep were divided into four groups: the lean and fat groups were given saline, while the lean-R and fat-R groups received resistin 1 h prior to euthanasia. Changes in adiposity influenced the lowering effect of resistin on the expression of LeptRa and VEGF system protein concentrations. Overall, both photoperiodic and nutritional signals influence the effects of MTS-leptin/roleptin and resistin-mediated leptin transport to the CNS via LeptRa. Resistin seems to be another adipokine involved in the adaptive/pathological phenomenon of leptin resistance in sheep.

## 1 Introduction

Leptin and resistin are crucial for managing lipid and glucose metabolism, inflammation, and immunity irregularities, with many of their effects being photoperiod-dependent (Biernat et al., 2018). Recently, attention has focused on the role of these two adipokines in the maintenance of energy balance in seasonally breeding species (Biernat et al., 2018; Zieba et al., 2020a). In temperate areas, the reproduction of sheep is primarily seasonal, depending mainly on the length of the day, and the phenomenon of reversible leptin resistance during long days (LD) allows these animals to live in harsh environments and store energy that they will be able to use during periods of reduced food availability. In autumn and winter, sheep are physiologically sensitive to leptin, and their appetite adjusts in proportion to their nutritional status (Szczesna and Zieba, 2015). In spring and summer, when food is plentiful and more readily available, sheep show an increased appetite and seem to be resistant to the high concentrations of leptin that result from increased adiposity. Leptin resistance can either be a pathological state, for example, in diet-induced obesity (DIO) or in a hyperleptinemia state, or it can be an adaptive response and physiological phenomenon to allow shifts in the body weight set point, for example, in seasonal animals. The primary cause of leptin resistance has not yet been identified, but it is presumed to be due to (1) a decrease in leptin transport from the bloodstream to the hypothalamus (termed peripheral resistance); (2) desensitization of leptin receptors (termed central resistance); (3) suppression of the long isoform of the leptin receptor (LepRb)-associated signaling pathways by negative regulators such as suppressor of cytokine signaling 3 (SOCS3; also termed central resistance); (4) hyperleptinemia associated with increasing fat depots and elevated circulating leptin concentrations; or (5) hypothalamic inflammation; obesity is a state of chronic low-grade inflammation and endoplasmic reticulum stress that drives the expression of negative regulatory molecules, such as SOCS3, which may cause the development of leptin resistance in hypothalamic neurons, particularly in arcuate nuclei (ARC; termed cellular leptin resistance; Szczesna and Zieba, 2015; Zhao et al., 2019).

The blood–brain barrier (BBB) is a key player in adipokine signaling from the periphery to the central nervous system (CNS). The results of our previous study demonstrate that photoperiodic and nutritional signals influence the effects of resistin on leptin transport to the CNS via the LeptRa isoform and that leptin action in the hypothalamus via the LeptRb isoform and resistin is involved in the adaptive/pathological phenomenon of leptin resistance in sheep, affecting the expression of SOCS3 (Król et al., 2007; Zieba et al., 2019; Biernat et al., 2021). To act centrally, leptin from circulating blood must first enter the brain, thereby passing through the blood-cerebrospinal fluid barrier (BCSFB) at the choroid plexus (ChP) and/or the BBB at the cerebral endothelium in the CNS. The LeptRa is predominantly found in the ChP and BBB; however, a high concentration of leptin in the blood could saturate LeptRa, decreasing leptin transport and leading to leptin resistance (Myers et al., 2010). Located within the cerebral ventricles, the ChP bridges the blood and CSF. The ChP and CSF are integral in the neuroendocrine regulatory systems that govern seasonal reproduction in animals and particularly in sheep, and ChP operation is strongly guided by photoperiods (Szczepkowska et al., 2020). Notably, protein levels in the ovine ChP related to tight junctions are higher during short days (SD) than LD, leading to differences in the permeability of the BCSFB and thus the ability of molecules to access the CSF from the periphery. For instance, the transport of steroid hormones from the blood to the CSF is significantly more prevalent during LD in sheep (Thiery and Malpaux, 2003; Thiery et al., 2006). This could also be due to changes in CSF molecule concentration or dilution, as CSF turnover is higher during SD (Thiéry et al., 2009). The functionality of fenestrated capillaries of ChP is regulated by vascular endothelial growth factor (VEGF); hence, a higher expression of VEGF receptor 2 (VEGFR2) under SD allows for photoperiod-related changes in the ChP capillaries (Szczepkowska et al., 2012). In ovine ChP, two isoforms are expressed, VEGFA_120_ and VEGFA_164_ (Szczepkowska et al., 2012). Nutritional status most likely modulates the plasticity of the blood-CSF barriers in this region. Flawed leptin transport to the CNS plays a major role in obesity development (Szczesna and Zieba, 2015). Later studies highlighted a phase when DIO animals resisted peripheral leptin but responded to central leptin administration. This suggested that leptin was inadequately crossing the BBB and that BBB resistance was the main driver in the earlier stages of leptin resistance.

Leptin administration has shown potential in aiding weight loss efforts, and research continues to explore ways to enhance leptin penetration into the brain to increase its therapeutic efficiency. Several proteins, such as TAT-leptin (Ahima et al., 1997), P85-leptin (Cheung et al., 1997), and MTS-leptin (Tomimatsu et al., 1997), have been identified and used as proteins capable of crossing the BBB independent of the leptin BBB transporter, leading to reduced food consumption and weight loss (Gertler and Solomon, 2013). Adam and Findlay, using the ovine model of obesity, which was characterized by increased body weight (BW) and external adiposity score and relatively increased ‘bad’ (low-density lipoprotein—LDL) cholesterol concentrations in circulating plasma (Adam and Findlay, 2010), support the hypothesis that the loss of the anorectic properties of central leptin associated with obesity is attributable to a decreased efficiency of blood–brain leptin transport and not to leptin insensitivity within the hypothalamus.

To test the hypothesis that photoperiod and nutritional status (fasted vs. fed state) modulate leptin entry to CNS, the changes in the mRNA levels of LeptRa and VEGF system (VEGFA and VEGFR2) in leptin and MTS-leptin-treated sheep were determined. The different brain areas were investigated: hypothalamic nuclei (ARC, the ventro- and dorsomedial nuclei—VMH/DMH, and the preoptic area—POA), anterior pituitary (AP), and ChP.

Resistin is a small protein with a mass of 12.5 kD that enters the brain via the BBB and is involved in leptin resistance (Adam and Findlay, 2010; Arnoldussen et al., 2014; Zieba et al., 2019). Two different metabolic/nutritional sheep models (lean and fat) were used to verify the hypothesis that alterations in BW affect resistin-mediated effects on the mRNA of LeptRa and the VEGF system.

Considering the above factors, the research was divided into two experiments; because sheep are seasonal animals, in the first experiment, we accounted for the length of the day, and the studies were carried out during long and short photoperiods. The second experiment was conducted over longer days when leptin resistance existed and highlighted the modulating role of nutritional status on ChP responses.

## 2 Materials and methods

The Second Local Ethics Committee on Animal Testing in Krakow, Poland, approved all the procedures conducted on the animals during these experiments (Protocols No. 16/2016 and 109/2018).

### 2.1 Animals

The research was conducted at the Experimental Station of the Department of Animal Nutrition, Biotechnology and Fisheries of the University of Agriculture in Krakow (longitude: 19°57 E, latitude: 50°04 N). A total of 80 female Polish Longwool sheep, bred with strong reproductive seasonality, were selected for the experiments. Ewes were 2 to 3 years of age, weighed 60 ± 5 kg, and were kept under natural photoperiodic and thermoperiodic conditions in individual boxes. Before the study began, ewes were bilaterally ovariectomized as described by Biernat et al. (2018) to eliminate the variability resulting from the differences in the concentration of sex hormones and were implanted subcutaneously with a silastic estradiol implant. In this study, the mean estradiol concentration in implanted sheep was 3.35 ± 0.45 pg/mL. Hormonal implants provide a constant negative feedback of steroids without complications related to ovarian cycling. Ovariectomized females and castrated males treated with steroids have been proven to be well-established models for studying neuroendocrine responses (Zieba et al., 2004). A body condition score of sheep was calculated (BCS = 3, on a scale from 1 to 5; Russel et al., 1969).

### 2.2 Experimental design

In both seasons, during SD and LD, sheep were often placed on wooden carts, as previously reported, to reduce stress during the experiment (Biernat et al., 2018). The carts allowed sheep to stand or lie down freely during the sampling period and to make eye contact with other animals. On the morning of the day of each experiment, the ewes were cannulated with jugular vein catheters (Central and Peripheral Venous Catheters, Careflow™, Argon, Billmed, Warsaw, Poland) for blood sampling collection.

### 2.3 Animal treatments

#### 2.3.1 Experiment 1: the effects of photoperiod and MTS-leptin on hormone plasma concentrations and the expression of LeptRa and the VEGF system (VEGFA and VEGFR2) in selected brain areas

In LD in May and SD in November, ewes (*n* = 30/season) were randomly divided into two groups: (1) F–sheep fed twice a day with 0700 and 1600 diets to provide 100% of Institut National De La Recherche Agronomique (2007)recommendations for maintenance [INRA], with constant access to water (*n* = 15/season) and (2) NF—ewes fasted for 72 h with free access to water (*n* = 15/season). Furthermore, in each season, sheep from the F and NF groups were randomly assigned to one of three treatment groups (*n* = 5/group/season). Treatment groups (Table 1) were as follows: (1) C-F, normal-fed sheep intravenously (i.v.) treated with saline (*n* = 5), (2) L-F, normal-fed sheep, i.v. treated with recombinant ovine leptin (roleptin—0.5 μg/kg of BW; *n* = 5), (3) MTS-F, normal-fed sheep, i.v. treated with MTS-leptin (0.5 μg/kg BW; *n* = 5), (4) C-NF, fasted sheep i.v. treated with saline (*n* = 5), (5) L-NF, fasted sheep i.v. treated with roleptin (0.5 μg/kg of BW; *n* = 5), and (6) MTS-NF, fasted sheep i.v. treated with MTS-leptin (0.5 μg/kg BW; *n* = 5). The roleptin and MTS-leptin doses were chosen based on our previous study (Zieba et al., 2008) and preliminary experiments and were purchased from Ansh Labs LLC (Webster, TX, United States). At the beginning of the experiment, saline/roleptin/MTS-leptin was injected through the catheter, and 1 h later, blood was collected through the same catheter. Blood samples (5 mL) were dispensed into test tubes containing 150 μL of a solution containing heparin (10,000 IU/mL) and 5% (w/v) EDTA (ethylene diamine tetraacetic) and placed on ice immediately. Plasma was separated by centrifugation and stored at −20°C until analyses. The ewes were humanely euthanized by captive bolt stunning after blood collection, and brains with the infundibulum remaining intact were rapidly removed from all ewes and frozen on dry ice. Samples of the ARC, VMH/DMH, POA, ChP, and AP were aseptically isolated from the ewes 10–15 min postmortem. Thus, the selected brain tissues were collected by removing a tissue block encompassing the hypothalamic–infundibular complex, followed by transection into two halves. An anterior coronal cut was made ~3–5 mm rostral to the optic chiasm, and a posterior coronal cut was made, encompassing approximately one-third of the mamillary body. A longitudinal cut parallel to the ventral surface of the brain ~2–3 cm dorsal to the anterior commissure followed. At the same time, the pituitary was harvested from the *sella turcica*. Isolated tissues were frozen immediately on dry ice for storage at −80°C. To eliminate the possibility of contamination by transferring tissue between samples, separate sterile tools were used to dissect each area. Tissue samples were rinsed in phosphate-buffered saline (PBS; Laboratory of Vaccines, Lublin, Poland), snap-frozen in liquid nitrogen, transferred and then stored at −80°C until analysis.

**Table 1:** List of experimental group names and acronyms from Experiment 1 and list of factors (saline, leptin, MTS-leptin) administered intravenously in each group, respectively.

Experiment	Experimental groups		Treatment (5.0 mL) intravenously	Number of animals
	Acronym	Full name	Intravenously	
1	C-F	Control-Fed	Saline	10
1	L-F	Leptin-Fed	Leptin 0.5 μg/kg BW	10
1	MTS-F	MTS Leptin- Fed	MTS-leptin 0.5 μg/kg BW	10
1	C-NF	Control-Non-Fed (Fasted)	Saline	10
1	L-NF	Leptin-Non-Fed (Fasted)	Leptin 0.5 μg/kg BW	10
1	MTS-NF	MTS Leptin-Non-Fed (Fasted)	MTS-leptin 0.5 μg/kg BW	10

#### 2.3.2 Experiment 2: the effect of BW and resistin treatment on LeptRa expression and the VEGF system in choroid plexus in sheep

A total of 20 animals were randomly assigned into two groups: food-restricted (Lean, *n* = 10) and a high-energy diet developed to increase BW (Fat, *n* = 10), which was achieved with an altered diet over 5 months. When food restriction was applied for extended periods, the sheep were housed in groups, and the lean animals received approximately 400 g of pasture hay/day supplemented with straw *ad libitum* as a filler. At the beginning of the experiment, the average BCS was 3.1 ± 0.3 (Russel et al., 1969). The objective of the adiposity score was to alter the BCS of animals to 2. The fat animals received pasture hay *ad libitum* plus a dietary supplement of approximately 1 kg lupin grain/animal/week, which increased adipose deposition. The animals were weighed every 2 weeks, and target weights were reached within 4 months, after which the diets were maintained for another month as described in a paper by Zieba et al. (2020b). The nutrient requirements for sheep and the diets’ exact compositions were determined based on INRA. The day before the experiment, the sheep were assigned to one of four treatment groups (*n* = 5/group/treatment). The experimental groups were as follows: the Lean (*n* = 5) and Fat (*n* = 5) groups were administered saline (5.0 mL), and the Lean-R (*n* = 5) and Fat-R (*n* = 5) groups were i.v. administered one dose of 5.0 μg/kg BW (5.0 mL) recombinant bovine resistin (rbresistin). At the beginning of the experiment, saline/rbresistin was injected through the catheter, and 1 h later, blood was collected through the same catheter. Blood samples (5 mL) were dispensed into test tubes containing 150 μL of a solution containing heparin (10,000 IU/mL) and 5% (w/v) EDTA and placed on ice immediately. After blood collection, the animals were humanely euthanized by captive bolt stunning. The ChPs were collected using the same methodology as described in Experiment 1. ChP samples were rinsed in PBS, snap-frozen in liquid nitrogen, and then stored at −80°C until analysis.

### 2.4 Hormone assays

Estradiol concentrations were determined using commercially available enzyme immunoassay (EIA) kits (DRG Instruments GmbH, Marburg, Germany) according to the manufacturer’s instructions. The inter- and intraassay precision values exhibited CVs of 3.46% and 2.4%, respectively. The assay sensitivity was 1.9 pg/mL. Plasma leptin concentrations were determined using a highly specific ovine leptin RIA with the double-antibody method, employing a specific, high-affinity rabbit antibody generated against recombinant ovine leptin and anti-rabbit-γ-globulin antisera and a recombinant ovine leptin standard, as described by Delavaud et al. (2000). The intra- and interassay coefficients of variation of the leptin assay were 2.4 and 10.7%, respectively, and the assay sensitivity was 0.3 ng/mL.

### 2.5 Determination of protein (VEGFA and VEGFR2) concentration in ChPs

The concentrations of the VEGFA and VEGFR2 proteins in the ChPs were determined using an ELISA kit (Cloud-Clone Corp., United States) designed and validated for sheep. The tissues were homogenized in 1 mL of cold lysis buffer (Cloud-Clone Corp., United States) containing a 1% protease inhibitor cocktail (Sigma Aldrich, Poznan, Poland) using a Bead Ruptor 12-bead mill homogenizer (Omni International, Inc., Kennesaw, GA, United States). The suspension was subjected to two freeze–thaw cycles to further break the cell membranes. Homogenates were then centrifuged for 5 min at 10,000 × g at 4°C. The supernatants were aliquoted and stored until assay at −80°C. The concentration of the isolated total protein was measured using bicinchoninic acid (Sigma Aldrich, Poznan, Poland). ELISAs were performed according to the manufacturer’s instructions. The absorbance measurements at 450 nm were performed using an ELx800 reader (BioTek, Biomedica, Piaseczno, Poland). The sensitivity of the VEGFA assay was 5.7 pg/mL, and the intra- and interassay coefficients of variation (CVs) were <10% and <12%, respectively. The sensitivity of the VEGFR2 assay was 0.262 ng/mL, and the intra- and interassay CVs were <10% and <12%, respectively.

### 2.6 Hormone and protein statistical analysis

All hormone and protein data are presented as the mean ± SEM. Data analysis was performed by a series of two-way ANOVAs using SigmaPlot® statistical software (version 11.0; Systat Software Inc., Richmond, CA, United States), preceded by Grubb’s test to identify outliers. All data sets with failed tests of normality and/or equal variance were transformed as natural logarithms. If the main effects or their interactions were significant, the Holm–Sidak test was used as a post-ANOVA test to compare individual means. A *p*-value < 0.05 was considered to indicate statistical significance.

### 2.7 Molecular analysis

The mRNA expression of LeptRa was measured using real-time PCR. Tissue homogenization was performed with a rotor-stator homogenizer (Omni TH, Omni International, Inc., Kennesaw, GA, United States) and single-use tips (Soft Tissue Omni Tip Plastic Homogenizing Probes, Omni International, Inc., Kennesaw, GA, United States). Total RNA was isolated using TRIzol reagent (Ambion Inc., Austin, TX, United States) following the manufacturer’s protocol. The samples were incubated at 42°C for 2 min with gDNA Wipeout Buffer (QuantiTect Reverse Transcription Kit; Qiagen, Hilden, Germany) to eliminate contamination of genomic DNA. Subsequently, to obtain samples of cDNAs by reverse transcription, isolates of RNA (1.0 μg) were incubated with Quantiscript reverse transcriptase and RT primer mix (QuantiTect Reverse Transcription Kit; Qiagen, Hilden, Germany) at 42°C for 15 min. The reaction was terminated by heating the samples to 94°C for 3 min. Each cDNA was amplified in triplicate using an Applied Biosystems 7300 Real-Time PCR System, TaqMan Gene Expression Master Mix, specific primers (900 nM) corresponding to the target/reference genes (Sequence Detection Primers), and specific probes (250 nM) corresponding to the target/reference genes (TaqMan MGB Probes) supplied by Life Technologies (Foster City, CA, United States). The primers and probes were designed using Primer Express software v. 2.0 (Applied Biosystems; Foster City, CA, United States) and are described in Table 2. The thermal profile for real-time PCR was as follows: (1) 50°C for 2 min—initial incubation; (2) 95°C for 10 min—activation of polymerase; (3) 40 cycles for denaturation (95°C for 15 s) and annealing/elongation (60°C for 60 s). The collected data were recorded with Applied Biosystem 7300 Real-Time PCR System SDS software.

**Table 2:** Sequences of oligonucleotides used as primers and probes to analyze the mRNA expression of cyclophilin (CPH; reference gene), the short form of the leptin receptor (LeptRa; target gene) in sheep.

Gene	Primer sequence (5′-3′)	Probe sequence (5′-3′)	Amplicon size	Gen bank accession number
CPH	CGGCTCCCAGTTCTTCATCA ACTACGTGCTTCCCATCCAAA	FAM-CGTTCCGACTCCGC-MGB	64 bp	D14074
LeptRa	TCAAAGTATGTCCGTTCTCTTCTG TCTTATTGCTTGGAACATTGTCA	FAM-TGTTTTGGGAAGATGTTC-MGB	131 bp	NM_001009763.1

#### 2.7.1 Molecular data analysis

The expression levels were calculated using relative quantification (RQ) analysis, and the results are expressed as a function of the threshold cycle (Ct), which is a value corresponding to the fractional PCR cycle number at which the fluorescent signal reaches the detection threshold. The data were analyzed using the 2^−ΔΔCt^ method, and Ct values were converted to fold-change RQ values. The RQ values from each gene were used to compare target gene expression across all groups. The mean mRNA expression levels for the target genes in each sample were standardized against the expression of a reference gene (cyclophilin; CPH) and expressed relative to the calibrator sample. The variation in the Ct values for CPH among the treatment groups was not significant (*p* > 0.05).

The mean ΔCt value for tissue collected from the control group was adopted as a calibrator to compare the changes in target gene expression between all the treatment groups in the indicated season. Differences in the means were compared with SigmaPlot statistical software (version 11.0; Systat Software Inc., Richmond, CA, United States) using all pairwise multiple comparison procedures (Tukey test), preceded by the determination of a significant *F* value. Differences were considered statistically significant when *p* < 0.05.

## 3 Results

### 3.1 The effects of photoperiod and MTS-leptin on hormone plasma concentrations and the expression of LeptRa and the VEGF system (VEGFA and VEGFR2) in selected brain areas

#### 3.1.1 Hormone plasma concentrations

**Figure 1:**
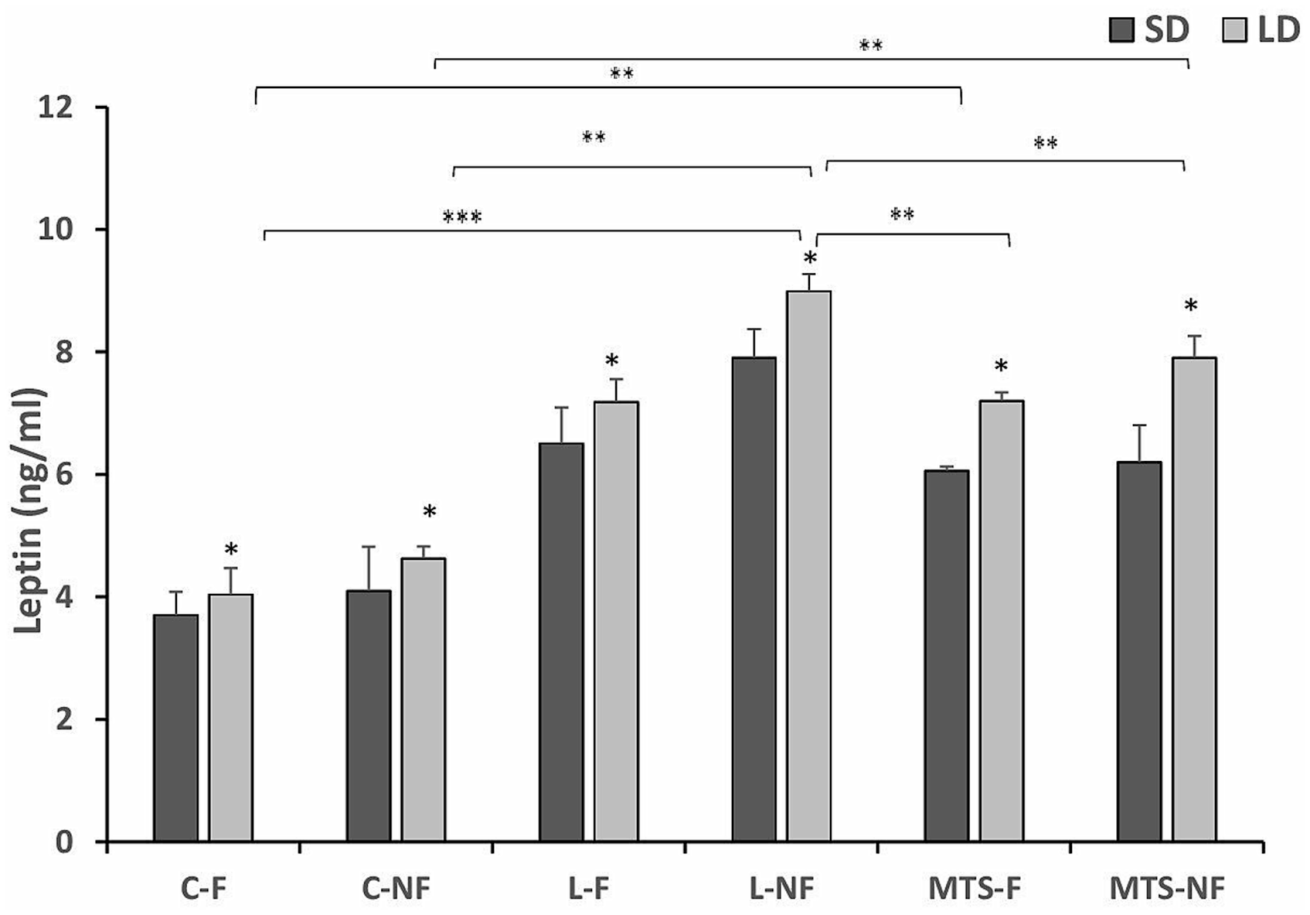
Plasma leptin concentrations. Mean concentrations of circulating (±SEM) leptin in saline (C) and recombinant ovine leptin-(L) and MTS-treated groups in fed (F) and fasted (N-F) animals during the long (LD) and short (SD) photoperiods. ^*^p < 0.05, ^**^p < 0.01, and ^***^p < 0.001 denote differences between groups.

The mean circulating concentration of estradiol (mean ± SEM) was 3.35 ± 0.45 pg/mL. Generally, leptin concentrations in all groups were higher during the LD season than during the SD season (*p <* 0.05). The concentrations of leptin were higher in the group of L-NF ewes during LD season almost 2-fold higher compared to C-NF and C-F (*p <* 0.01), the treatment with MTS-leptin increased leptin concentration in LD season 2-fold in MTS-NF and 1.5-fold in MTS-F groups compare to Controls (*p <* 0.01). There were differences (*p <* 0.05) between the MTS-F and MTS-NF groups and the L-NF group (Figure 1).

#### 3.1.2 Expression of LeptRa mRNA in selected brain areas

**Figure 2:**
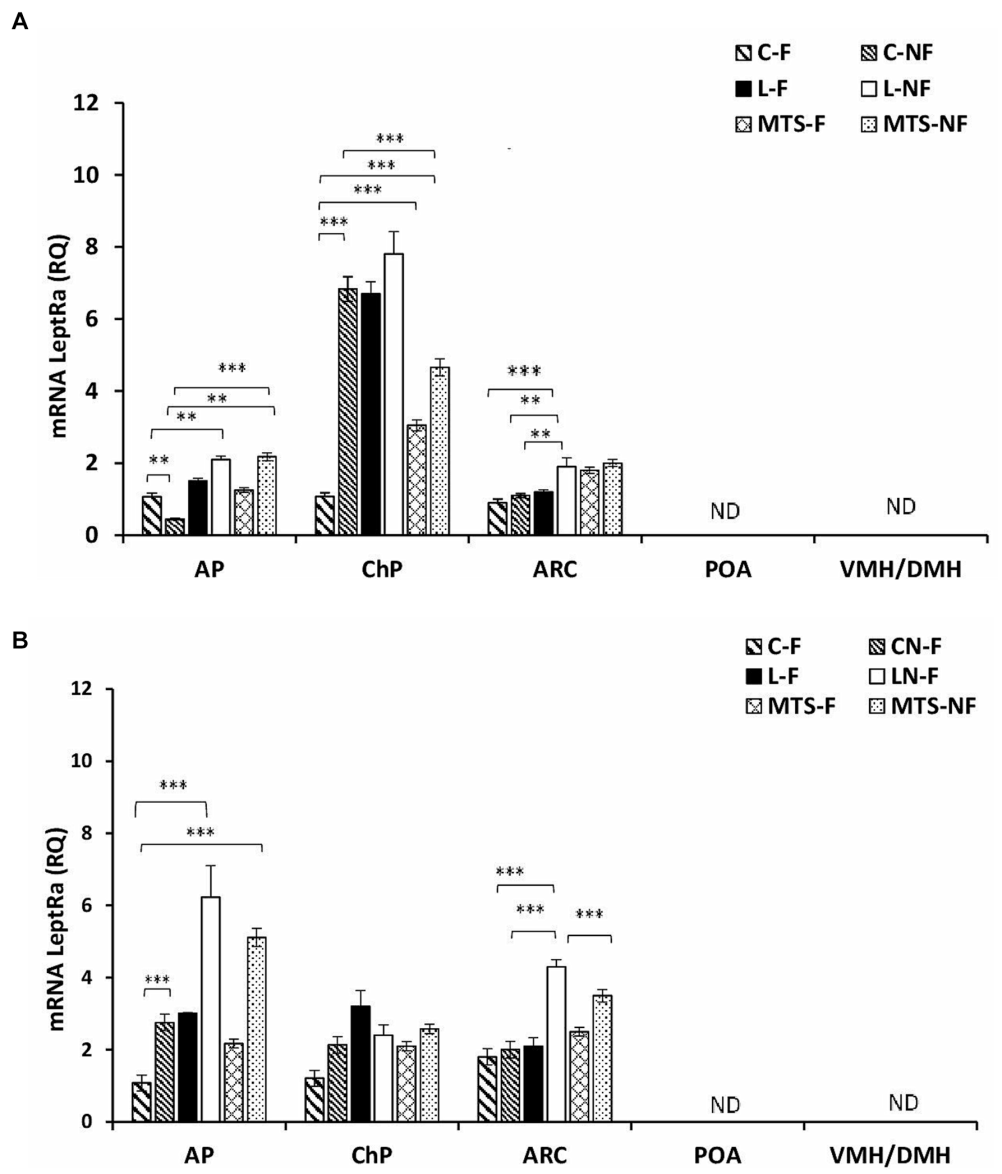
Leptin receptor expression. The mean expression (±SEM) of the short form of leptin receptor (LeptRa) mRNA in ovine anterior pituitary (AP), arcuate nucleus (ARC), preoptic area (POA) and ventro- and dorsomedial nuclei (VMH/DMH) collected during short-day (SD)—panel **(A)** and long-day (LD)—panel **(B)**, photoperiods. The expression of LeptRa mRNA is reported in arbitrary units (RQ) relative to cyclophilin mRNA expression and expressed relative to the calibrator sample. ^**^p < 0.01 and ^***^p < 0.001 denote differences between groups.

Transcripts of LeptRa were detected at varying levels in the selected brain tissues, namely, the AP, ChPs, and ARC; however, no detection was noted in the POA and VMH/DMH nuclei during either the SD or LD seasons (Figures 2A,B). In the AP, during the SD season, the expression of LeptRa increased in samples collected from the L-NF and MTS-NF groups by approximately 0.5-fold (*p <* 0.01) and 2.0-fold (*p <* 0.01), respectively, compared to the control-fed group. A significant increase (*p <* 0.01) was found between the treatment groups during LD (Figure 2B). The mRNA transcript level of LeptRa increased in fasted groups treated with saline—2.5-fold (*p <* 0.001), roleptin—6-fold (*p <* 0.001), and MTS-leptin; 5-fold (*p <* 0.001) compared to Control fed ewes (Figure 2B).

During the SD season, the LeptRa transcripts in the ChPs increased 7-fold (*p <* 0.001) in the C-F and LF groups and 8-fold (*p <* 0.001) in the L-NF group compared to the control-fed sheep (Figure 2A). An increase was also observed in MTS groups: 2-fold (*p <* 0.001) in MTS-F and 4-fold (*p <* 0.001) in MTS-NF group compared to C-F. During the LD season, within the ChR, there were no significant differences between treated groups. For the ARC, during the SD season, a 0.5-fold (*p <* 0.001) increase in the mRNA levels of LeptRa was noted in the L-NF group compared to the L-F and Control groups (*p <* 0.01), and in the LD season, a 1.5-fold increase was noted (*p* < 0.001) between the L-NF and C-NF and C-F groups. A decrease (*p* < 0.001) was noted between the L-NF and MTS-NF groups.

**Figure 3:**
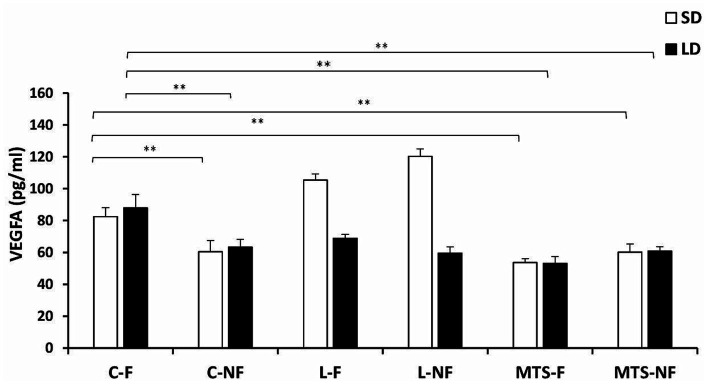
VEGFA protein concentration. The mean protein concentration (±SEM) of VEGFA in ovine choroid plexus (ChP) after saline (C) and recombinant ovine leptin-(L) and MTS-treated groups in fed (F) and fasted (N-F) animals during the long (LD) and short (SD) photoperiods. ^**^p < 0.01 denotes differences between groups.

**Figure 4:**
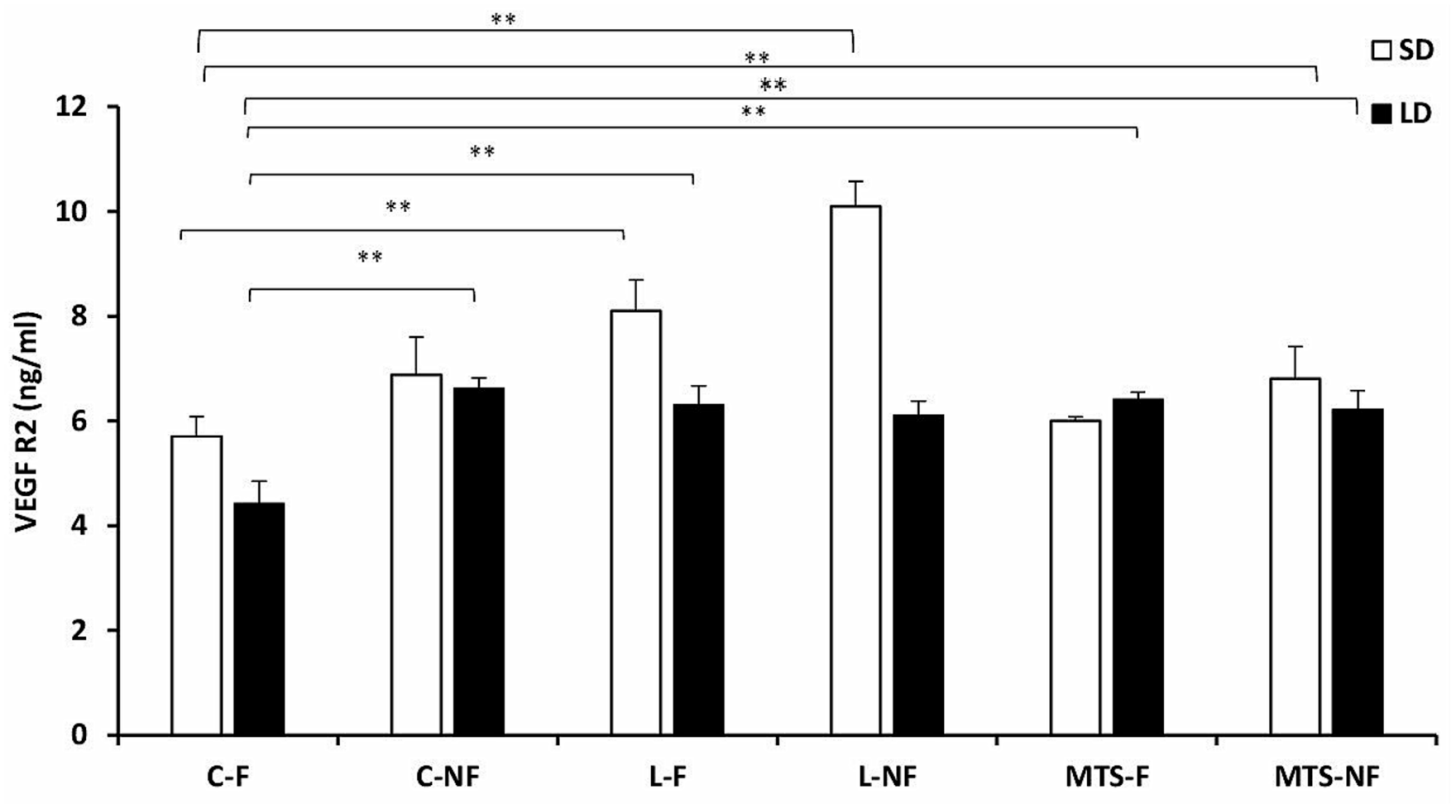
VEGFR2 protein concentration. The mean protein concentration (±SEM) of VEGFA in ovine choroid plexus (ChP) after saline (C) and recombinant ovine leptin-(L) and MTS-treated groups in fed (F) and fasted (N-F) animals during the long (LD) and short (SD) photoperiods. ^**^p < 0.01 denotes differences between groups.

During the SD and LD seasons, VEGFA concentrations were lower in C-NF (*p <* 0.01), MTS-F (*p <* 0.001), and MTS-NF (*p <* 0.01) sheep than in control-fed ewes (Figure 3). There was an increase (*p <* 0.01) in the protein concentration of VEGFA in the L-F and L-NF groups compared to the C-F and C-NF groups in the SD season. For VEGFR2 concentrations, during the SD season, an increase was noted between the C-F and (*p <* 0.001) L-F groups, (*p <* 0.01) L-NF, and (*p <* 0.01) MTS-NF groups. In the LD photoperiod, changes were noted between C-F and (*p <* 0.01) L-F, (*p <* 0.01) L-NF, (*p <* 0.01) MTS-F, and (*p <* 0.01) MTS-NF (Figure 4).

### 3.2 The effect of BW and resistin treatment on LeptRa expression and the VEGF system in choroid plexus in sheep

Initial BW did not differ between treatment groups. However, final body weight and average daily gain were greater (*p* = 0.04) in fat sheep than in lean sheep. There were significant (*p* < 0.01) differences in BW between the lean and fat groups, with the lean ewes weighing 41.2 0 ± 0.92 and the fat ewes weighing 78.1 ± 1.78 kg. The injection of 5.0 μg/kg BW rbresistin increased (*p <* 0.05) circulating resistin concentrations in the fat and lean-R sheep compared to the nontreated lean ewes. Significant differences (*p <* 0.05) were noted between the fat and fat-R groups and between the lean-R and fat-R groups (*p <* 0.05). Circulating concentrations (mean ± SEM) of leptin after rbresistin treatment were higher (*p* < 0.01) in fat and lean-R sheep than in lean sheep. Recombinant bovine resistin increased (*p* < 0.001) endogenous leptin concentrations in the fat-R group vs. the fat and lean-R experimental groups of sheep.

**Figure 5:**
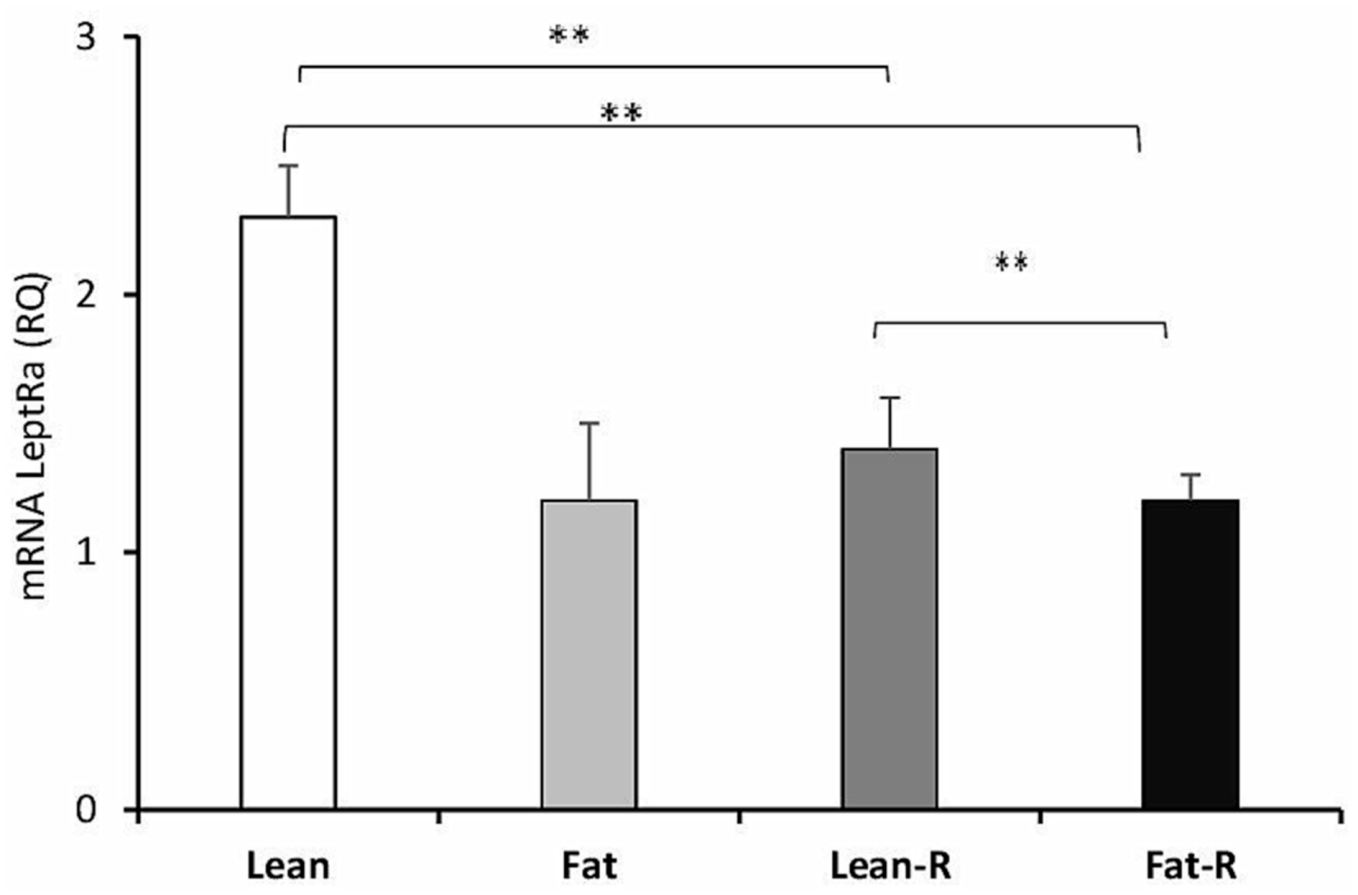
Leptin receptor expression. The mean expression (±SEM) of the short form of leptin receptor (LeptRa) mRNA in ovine choroid plexus in nontreated (Lean and Fat) animals and animals treated with recombinant bovine resistin (Lean-R and Fat-R) after 5 months of body weight alternation (*n* = 5 per group). The expression of LeptRa mRNA is reported in arbitrary units (RQ) relative to cyclophilin mRNA expression and expressed relative to the calibrator sample. ^**^p < 0.01 denotes differences between groups.

The expression of LeptRa was 2-fold lower (*p* < 0.01) in the lean-R group and Fat-R group (*p* < 0.01) than in the Lean group. There were significant differences (*p* < 0.01) between the lean-R and fat-R ewes (Figure 5).

**Figure 6:**
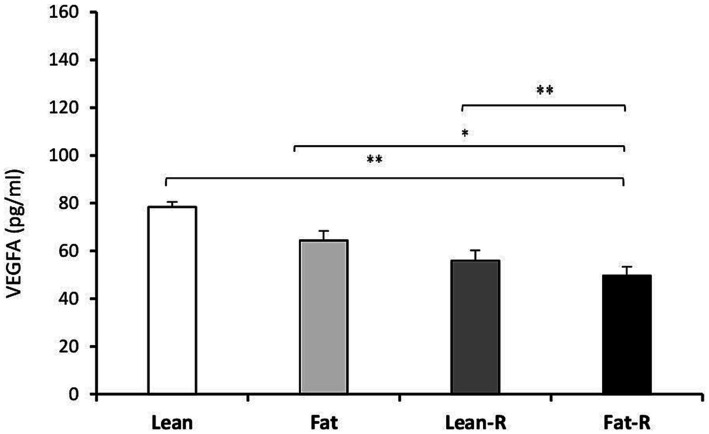
VEGFA protein concentration. The mean protein concentration (± SEM) of VEGFA in ovine choroid plexus in nontreated (Lean and Fat) animals and animals treated with recombinant bovine resistin (Lean-R and Fat-R) after 5 months of body weight alternation (*n* = 5 per group). ^*^p < 0.05 and ^**^p < 0.01 denote differences between groups.

**Figure 7:**
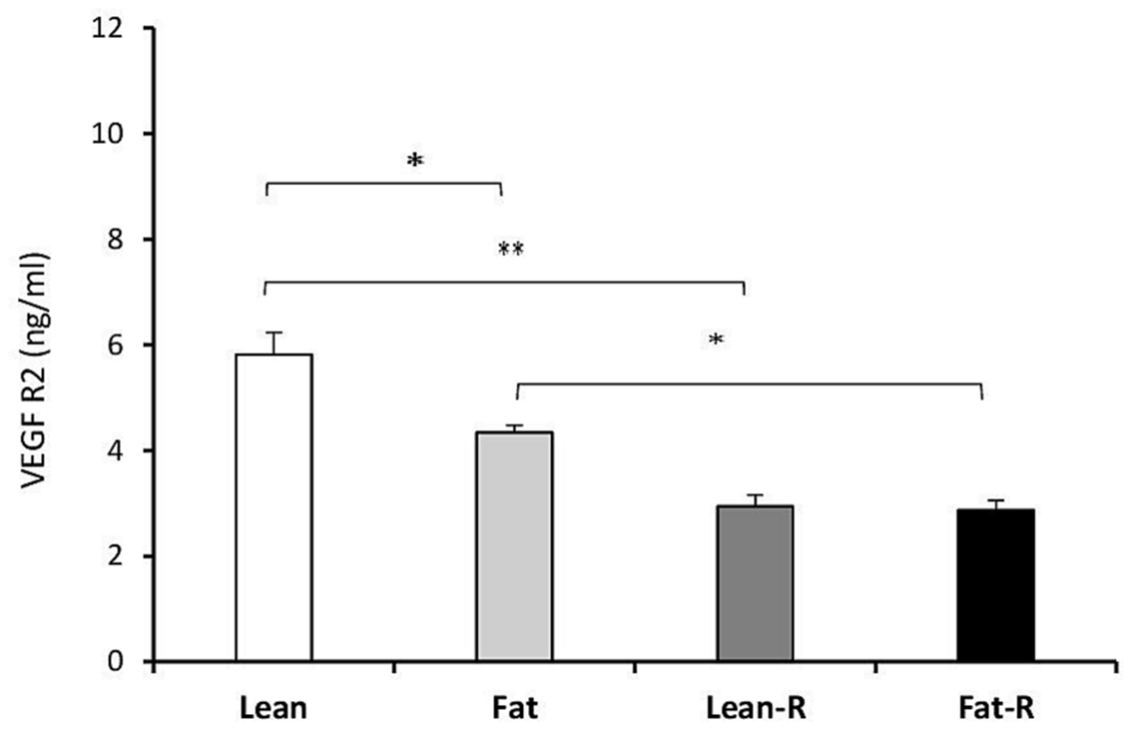
VEGFR2 protein concentration. The mean protein concentration (± SEM) of VEGFR2 in ovine choroid plexus in nontreated (Lean and Fat) animals and animals treated with recombinant bovine resistin (Lean-R and Fat-R) after 5 months of body weight alternation (*n* = 5 per group). ^*^p < 0.05 and ^**^p < 0.01 denote differences between groups.

Considering the VEGF system, the concentrations of VEGFA in the Lean-R and Fat-R groups decreased compared to (*p* < 0.01) those in the Lean, (*p* < 0.05) Fat and Fast-R groups (*p* < 0.01; Figure 6). The concentrations of VEGFR2 were lower in the Lean-R group (*p* < 0.01) than in the Lean (*p* < 0.01) group (Figure 7). In fat sheep, the concentration of VEGFR2 was lower (*p* < 0.05) than that in lean ewes but higher (*p* < 0.05) compared to Fat-R group.

## 4 Discussion

Leptin transport into the brain appears to be a limiting step in the modulation of its central effects, and it participates in leptin resistance in humans, rodents, and seasonal sheep (Caro et al., 1996; El-Haschimi et al., 2000; Zieba et al., 2020a). The results of the experiments showed for the first time the effect of MTS-leptin/roleptin on the expression of LeptRa, VEGFA, and VEGFR2 in selected brain regions: AP, ChPs, and ARC, in the context of nutritional status, resistin, and photoperiod. Leptin, as a therapeutic agent, has poor clinical efficacy because of its short circulating half-life, low potency, and poor solubility (Vilà et al., 1998). To improve the therapeutic profile of peptides, researchers chemically modified leptin and used TAT-leptin (Zhang et al., 2010) or Pluronic P85 (Price et al., 2010). In the present experiments, we used recombinant sheep MTS-leptin with one polypeptide chain of 157 amino acids (an additional 10 amino acids of the membrane translocating sequence Val-Leu-Leu-Pro-Val-Leu-Leu-Ala-Ala-Pro) derived from Kaposi virus and additional Ala acids at the N-terminus. The obtained molecular weight of MTS-leptin was ~17.5 kDa (Gertler and Solomon, 2013), and we compared its effect to the one we always use in research: recombinant ovine leptin with one polypeptide chain containing 146 amino acids having a molecular weight of ~16 kDa. The results showed that the nutritional status of sheep affected the concentration of endogenous leptin after treatment with MTS-leptin and/or recombinant oleptin, and the differences were dependent on photoperiod, with a weaker effect of MTS-leptin. The concentration of leptin was higher in fasted ewes during the LD season in all experimental groups, as we observed in our previous experiments on cattle and sheep (Zieba et al., 2003, 2008; Biernat et al., 2021). In prepubertal heifers, leptin gene expression and circulating leptin were markedly reduced in fasted (60 h) compared to fed females (Amstalden et al., 2000), and the leptin concentration was 2- or 3-fold lower in SD vs. LD sheep (Biernat et al., 2021).

Nutritional status (starved, fed, obese animals) can change the expression of many peptides and neuropeptides involved in the regulation of food intake and energy homeostasis. However, most animal experiments involve short-term fasting changes (Amstalden et al., 2000; Nagatani et al., 2000), and the effects of long-term changes in BW and adiposity still need to be fully investigated. The metabolic abnormalities connected with obesity can lead to the development of pathophysiological common conditions in humans, such as metabolic syndrome and insulin and leptin resistance (pathological status). We found no differences between the expression of mRNA for LeptRa and the VEGF system in lean and fat sheep. That was quite surprising, knowing that VEGF-mediated structural changes at the brain-hypothalamic barrier, by modulating the access of metabolic signals, e.g., insulin, leptin, ghrelin, and glucose to the ARC, and that the VEGF system plays an important role in the adaptive response to food deprivation (Langlet et al., 2013). In fasting mice, lower concentrations of blood glucose activate VEGFA expression in tanycytes, and VEGFA accumulation in the median eminence (ME) acts on endothelial VEGFR2 to promote the fenestration of capillary loops that reach the ARC (Coppari et al., 2005). The study by Langlet et al. (2013) was performed on nocturnal mice, and Szczepkowska et al. (2012) were working on sheep and observed VEGFR2 changes in ChP only during SD. As the second experiment was ultimately conducted during LD, these discrepancies may have resulted from the model on which the experiments were conducted: monogastric vs. ruminant species and photoperiod interaction. Although seasonal sheep are a very good research model with reversible leptin resistance throughout the year, they are ruminants that process and metabolize food differently than monogastric species and this should always be considered.

Importantly, differences between groups were noted after resistin treatment. The lowest expression of LeptRa in ChPs was observed in lean-R and fat-R sheep, and a lower concentration of VEGFA was noted in fat-R sheep, and a lower concentration of VEGFR2 was noted in lean-R and fat-R sheep compared to lean ewes. Our previous study demonstrated that in sheep, resistin can be added to a group of factors creating a leptin central insensitivity phenomenon during LDs, increasing SOCS3 expression and decreasing the expression of LeptRb in the hypothalamus (Zieba et al., 2019). The results suggest that this adipokine is involved in leptin translocation and leptin signaling to the most important hypothalamic nuclei responsible for energy resources, namely, the ARC (Cowley et al., 2001). We can conclude that leptin resistance status, as we observed in the previous and present studies, is accompanied by decreased leptin transport to the CNS with the resistin-lowering expression of the LeptRa and VEFG systems.

Although Szczepkowska et al. (2012) concluded that VEGFA may be involved in the photoperiodic plasticity of ChP capillaries, she found only slight changes in VEGFA expression between LD and SD photoperiods in estradiol-implanted and ovariectomized sheep (Skipor et al., 2010). Our study demonstrated in the same animal model that acute fasting (72 h) and photoperiod both modulated VEGFA and VEGR2 protein concentrations. The results indicate that high concentrations of VEGFA and VEGFR2 in the ChP of leptin-treated sheep not only during SD but also food deprivation contributed to a stronger effect. Taking the above information into account, as was indicated by Prevot et al. (2013), the VEGF system plays a very important role in adaptive changes of the barrier in the ME-ARC area, allowing for the rapid passage of metabolic signals during fasting or energy deficit.

The results of the first experiment indicate the role of acute fasting in the expression of LeptRa in the pituitary, ChP, and mediobasal hypothalamic arcuate nuclei. The hypothalamic regulation of energy homeostasis involves an interconnected neural network that contains specialized neurons located in the ARC, VMH, and DMH (Zieba et al., 2020b). Understanding how the networks of the hypothalamus respond to metabolic signals and integrate them to alter their output is still a subject of research in animal models, especially as unique as sheep exhibiting circannual reversible central leptin insensitivity (Zieba et al., 2008; Szczesna et al., 2011). Different responses of these tissues were observed in the leptin/MTS-leptin-treated sheep from the fed and/or fasted groups. We found no detection of the mRNA transcript of a short form of leptin receptor in the POA and VMH/DMH in either nutritional group. The different expression levels of LeptRa were noted in the ChP, ARC, and pituitary. This can be explained by the proximity of the ARC and ChP to the ME and the engagement of LeptRa in leptin translocation to the brain. The high expression of LeptRb in the POA and VMH/DMH demonstrated in a previous study confirms that LeptRb, which has a long intracellular domain, is the only isoform essential for intracellular signal transduction (Zieba et al., 2019).

Changes in the expression of LeptRa to acute fasting were demonstrated by a higher level in leptin/MTS-leptin-treated sheep and even in Control groups predominantly during SD when the hypothalamus is leptin sensitive and the transport of leptin is not disturbed. For the pituitary in this experiment, we observed the opposite response of tissue to leptin and MTS-leptin, as in many previous experiments (Zieba et al., 2008; Szczesna et al., 2011; Biernat et al., 2021). The results confirm that the hypothalamus and pituitary gland of sheep perceive the leptin signal differently and are sensitive/resistant to this adipokine in different photoperiods (Zieba et al., 2008; Szczesna et al., 2011). Very low expression of LeptRa in ChP in LD provides further evidence that leptin insensitivity, termed peripheral resistance, exists in sheep and joins other causes of leptin resistance demonstrated by us and other groups.

To conclude, MTS-leptin works like roleptin in seasonally breeding sheep. For the first time, it was demonstrated that both leptin and MTS-leptin modulate the expression of LeptRa and the VEGF system depending on photoperiod and acute fasting. Changes within ChP and ARC and the action of resistin on LeptRa/VEGFA/VEGFR2 in lean and fat ewes indicated that resistin is not only involved in central leptin resistance but also contributes to peripheral leptin resistance and actively reduced leptin transport to the CSN.

### Scope statement

To act centrally, leptin from circulating blood must first enter the brain, thereby passing through the blood-cerebrospinal fluid barrier at the choroid plexus via the short form of leptin receptor. Leptin transport depends on the nutritional status of the organism and photoperiod in seasonally breeding animals. The loss of the anorectic properties of leptin is most likely not caused by a single mechanism but rather results from a combination of many factors. However, the critical mechanisms that underlie this process remain unclear. Understanding the causes of the leptin-resistance phenomenon and central leptin insufficiency based on a large-animal model of unique potential (biannual reversible leptin resistance) contributes to the knowledge of the mechanisms underlying metabolic disturbance in both humans and animals. In our study, we used recombinant ovine leptin/modified MTS-leptin/bovine recombinant resistin to compare their effects in sheep and explore their engagement in physiological long-day and pathological (DIO) leptin resistance. Determination of the nature of the biological activity of leptin in the context of nutritional status and changes in VEGF and its receptor isoforms and short form of leptin receptor in the choroid plexus is the next step in understanding how leptin enters the brain.

## Data availability statement

The original contributions presented in the study are included in the article/supplementary material, further inquiries can be directed to the corresponding author.

## Ethics statement

The animal study was approved by the Second Local Ethics Committee on Animal Testing in Krakow, Poland, approved all the procedures conducted on the animals during these experiments (Protocols No. 16/2016 and 109/2018). The study was conducted in accordance with the local legislation and institutional requirements.

## Author contributions

DZ: Conceptualization, Funding acquisition, Project administration, Supervision, Writing – original draft, Writing – review & editing. WB: Conceptualization, Investigation, Writing – review & editing. MS: Investigation, Methodology, Conceptualization, Writing – review & editing. KK: Investigation, Methodology, Formal analysis, Writing – review & editing. TM: Methodology, Investigation, Writing – review & editing.

## References

[ref1] Adam C. L. Findlay P. A. (2010). Decreased blood–brain leptin transfer in an ovine model of obesity and weight loss: resolving the cause of leptin resistance. Int. J. Obes. (Lond) 34, 980–988. doi: 10.1038/ijo.2010.28, PMID: 20142821

[ref2] Ahima R. S. Dushay J. Flier S. N. Prabakaran D. Flier J. S. (1997). Leptin accelerates the onset of puberty in normal female mice. J. Clin. Invest. 99, 391–395. doi: 10.1172/jci119172, PMID: 9022071 PMC507811

[ref3] Amstalden M. Garcia M. R. Williams S. W. Stanko R. L. Nizielski S. E. Morrison C. D.. (2000). Leptin gene expression, circulating leptin, and luteinizing hormone pulsatility are acutely responsive to short-term fasting in prepubertal heifers: relationships to circulating insulin and insulin-like growth factor I(1). Biol. Reprod. 63, 127–133. doi: 10.1095/biolreprod63.1.127, PMID: 10859251

[ref4] Arnoldussen I. A. Kiliaan A. J. Gustafson D. R. (2014). Obesity and dementia: adipokines interact with the brain. Eur. Neuropsychopharmacol. 24, 1982–1999. doi: 10.1016/j.euroneuro.2014.03.002, PMID: 24704273 PMC4169761

[ref5] Biernat W. Kirsz K. Szczesna M. Zieba D. A. (2018). Resistin regulates reproductive hormone secretion from the ovine adenohypophysis depending on season. Domest. Anim. Endocrinol. 65, 95–100. doi: 10.1016/j.domaniend.2018.07.001, PMID: 30086525

[ref6] Biernat W. Szczęsna M. Kirsz K. Zieba D. A. (2021). Seasonal and nutritional fluctuations in the mRNA levels of the short form of the leptin receptor (LRa) in the hypothalamus and anterior pituitary in resistin-treated sheep. Animals 11:2451. doi: 10.3390/ani11082451 34438908 PMC8388769

[ref7] Caro J. F. Kolaczynski J. W. Nyce M. R. Ohannesian J. P. Opentanova I. Goldman W. H.. (1996). Decreased cerebrospinal-fluid/serum leptin ratio in obesity: a possible mechanism for leptin resistance. Lancet 348, 159–161. doi: 10.1016/s0140-6736(96)03173-x, PMID: 8684156

[ref8] Cheung C. C. Thornton J. E. Kuijper J. L. Weigle D. S. Clifton D. K. Steiner R. A. (1997). Leptin is a metabolic gate for the onset of puberty in the female rat. Endocrinology 138, 855–858. doi: 10.1210/endo.138.2.5054, PMID: 9003028

[ref9] Coppari R. Ichinose M. Lee C. E. Pullen A. E. Kenny C. D. McGovern R. A.. (2005). The hypothalamic arcuate nucleus: a key site for mediating leptin's effects on glucose homeostasis and locomotor activity. Cell Metab. 1, 63–72. doi: 10.1016/j.cmet.2004.12.004, PMID: 16054045

[ref10] Cowley M. A. Smart J. L. Rubinstein M. Cerdán M. G. Diano S. Horvath T. L.. (2001). Leptin activates anorexigenic POMC neurons through a neural network in the arcuate nucleus. Nature 411, 480–484. doi: 10.1038/35078085, PMID: 11373681

[ref11] Delavaud C. Bocquier F. Chilliard Y. Keisler D. H. Gertler A. Kann G. (2000). Plasma leptin determination in ruminants: effect of nutritional status and body fatness on plasma leptin concentration assessed by a specific RIA in sheep. J. Endocrinol. 165, 519–526. doi: 10.1677/joe.0.1650519, PMID: 10810316

[ref12] El-Haschimi K. Pierroz D. D. Hileman S. M. Bjørbaek C. Flier J. S. (2000). Two defects contribute to hypothalamic leptin resistance in mice with diet-induced obesity. J. Clin. Invest. 105, 1827–1832. doi: 10.1172/jci9842, PMID: 10862798 PMC378516

[ref13] Gertler A. Solomon G. (2013). Leptin-activity blockers: development and potential use in experimental biology and medicine. Can. J. Physiol. Pharmacol. 91, 873–882. doi: 10.1139/cjpp-2013-0012, PMID: 24117254

[ref14] Institut National De La Recherche Agronomique. (2007). Alimentation Des Bovins, Ovins et Caprins: Besoins Des Animaux, Valeurs Des Aliment. Paris, France: INRA.

[ref15] Król E. Tups A. Archer Z. A. Ross A. W. Moar K. M. Bell L. M.. (2007). Altered expression of SOCS3 in the hypothalamic arcuate nucleus during seasonal body mass changes in the field vole, *Microtus agrestis*. J. Neuroendocrinol. 19, 83–94. doi: 10.1111/j.1365-2826.2006.01507.x 17214870

[ref16] Langlet F. Levin B. E. Luquet S. Mazzone M. Messina A. Dunn-Meynell A. A.. (2013). Tanycytic VEGF-A boosts blood-hypothalamus barrier plasticity and access of metabolic signals to the arcuate nucleus in response to fasting. Cell Metab. 17, 607–617. doi: 10.1016/j.cmet.2013.03.004, PMID: 23562080 PMC3695242

[ref17] Myers M. G. Leibel R. L. Seeley R. J. Schwartz M. W. (2010). Obesity and leptin resistance: distinguishing cause from effect. Trends Endocrinol. Metab. 21, 643–651. doi: 10.1016/j.tem.2010.08.002, PMID: 20846876 PMC2967652

[ref18] Nagatani S. Zeng Y. Keisler D. H. Foster D. L. Jaffe C. A. (2000). Leptin regulates pulsatile luteinizing hormone and growth hormone secretion in the sheep. Endocrinology 141, 3965–3975. doi: 10.1210/endo.141.11.7762, PMID: 11089526

[ref19] Prevot V. Langlet F. Dehouck B. (2013). Flipping the tanycyte switch: how circulating signals gain direct access to the metabolic brain. Aging 5, 332–334. doi: 10.18632/aging.100557, PMID: 23667132 PMC3701106

[ref20] Price T. O. Farr S. A. Yi X. Vinogradov S. Batrakova E. Banks W. A.. (2010). Transport across the blood-brain barrier of pluronic leptin. J. Pharmacol. Exp. Ther. 333, 253–263. doi: 10.1124/jpet.109.158147, PMID: 20053933 PMC2846026

[ref21] Russel A. J. F. Doney J. M. Gunn R. G. (1969). Subjective assessment of body fat in live sheep. J. Agric. Sci. 72, 451–454. doi: 10.1017/S0021859600024874

[ref1002] Skipor J. Misztal T. Szczepkowska A. (2010). Thyroid hormones in the cerebrospinal fluid of the third ventricle of adult female sheep during different periods of reproductive activity. Pol. J. Vet. Sci. 13, 587–95. doi: 10.2478/v10181-010-0018-z 21370735

[ref22] Szczepkowska A. Kowalewska M. Krawczyńska A. Herman A. P. Skipor J. (2020). Photoperiod affects leptin action on the choroid plexus in ewes challenged with lipopolysaccharide-study on the mRNA level. Int. J. Mol. Sci. 21:7647. doi: 10.3390/ijms21207647, PMID: 33076568 PMC7589540

[ref23] Szczepkowska A. Wąsowska B. Gilun P. D. Lagaraine C. Robert V. Dufourny L.. (2012). Pattern of expression of vascular endothelial growth factor and its receptors in the ovine choroid plexus during long and short photoperiods. Cell Tissue Res. 350, 157–166. doi: 10.1007/s00441-012-1431-7, PMID: 22622803 PMC3462986

[ref24] Szczesna M. Zieba D. A. (2015). Phenomenon of leptin resistance in seasonal animals: the failure of leptin action in the brain. Domest. Anim. Endocrinol. 52, 60–70. doi: 10.1016/j.domaniend.2015.03.002 25863197

[ref25] Szczesna M. Zieba D. A. Klocek-Górka B. Misztal T. Stepien E. (2011). Seasonal effects of central leptin infusion and prolactin treatment on pituitary SOCS-3 gene expression in ewes. J. Endocrinol. 208, 81–88. doi: 10.1677/JOE-10-0282, PMID: 20962013

[ref26] Thiery J. C. Lomet D. Schumacher M. Liere P. Tricoire H. Locatelli A.. (2006). Concentrations of estradiol in ewe cerebrospinal fluid are modulated by photoperiod through pineal-dependent mechanisms. J. Pineal Res. 41, 306–312. doi: 10.1111/j.1600-079X.2006.00370.x 17014687

[ref27] Thiery J. C. Malpaux B. (2003). Seasonal regulation of reproductive activity in sheep: modulation of access of sex steroids to the brain. Ann. N. Y. Acad. Sci. 1007, 169–175. doi: 10.1196/annals.1286.017 14993051

[ref28] Thiéry J. C. Robel P. Canepa S. Delaleu B. Gayrard V. Picard-Hagen N.. (2009). Passage of progesterone into the brain changes with photoperiod in the ewe. Eur. J. Neurosci. 18, 895–901. doi: 10.1046/j.1460-9568.2003.02796.x, PMID: 12925015

[ref29] Tomimatsu T. Yamaguchi M. Murakami T. Ogura K. Sakata M. Mitsuda N.. (1997). Increase of mouse leptin production by adipose tissue after midpregnancy: gestational profile of serum leptin concentration. Biochem. Biophys. Res. Commun. 240, 213–215. doi: 10.1006/bbrc.1997.7638, PMID: 9367912

[ref30] Vilà R. Adán C. Rafecas I. Fernández-López J. A. Remesar X. Alemany M. (1998). Plasma leptin turnover rates in lean and obese zucker rats. Endocrinology 139, 4466–4469. doi: 10.1210/endo.139.11.6296, PMID: 9794453

[ref1001] Zhao S. Zhu Y. Schultz R. D. Li N. He Z. Zhang Z.. (2019). Partial leptin reduction as an insulin sensitization and weight loss strategy. Cell Metab. 30, 706–719.e6. doi: 10.1016/j.cmet.2019.08.005 31495688 PMC6774814

[ref31] Zieba D. A. Amstalden M. Morton S. Gallino J. L. Edwards J. F. Harms P. G.. (2003). Effects of leptin on basal and GHRH-stimulated GH secretion from the bovine adenohypophysis are dependent upon nutritional status. J. Endocrinol. 178, 83–89. doi: 10.1677/joe.0.1780083, PMID: 12844339

[ref32] Zieba D. A. Amstalden M. Morton S. Maciel M. N. Keisler D. H. Williams G. L. (2004). Regulatory roles of leptin at the hypothalamic-hypophyseal axis before and after sexual maturation in cattle. Biol. Reprod. 71, 804–812. doi: 10.1095/biolreprod.104.028548, PMID: 15128593

[ref33] Zieba D. A. Biernat W. Barć J. (2020a). The roles of leptin and resistin in reproduction and leptin resistance in sheep. Domest. Anim. Endocrinol. 73:106472. doi: 10.1016/j.domaniend.2020.106472, PMID: 32265081

[ref34] Zieba D. A. Biernat W. Szczesna M. Kirsz K. Barć J. Misztal T. (2020b). Changes in expression of the genes for the leptin signaling in hypothalamic-pituitary selected areas and endocrine responses to long-term manipulation in body weight and resistin in ewes. Int. J. Mol. Sci. 21:4238. doi: 10.3390/ijms21124238, PMID: 32545900 PMC7348850

[ref35] Zieba D. A. Biernat W. Szczesna M. Kirsz K. Misztal T. (2019). Hypothalamic-pituitary and adipose tissue responses to the effect of resistin in sheep: the integration of leptin and resistin signaling involving a suppressor of cytokine signaling 3 and the long form of the leptin receptor. Nutrients 11:2180. doi: 10.3390/nu11092180, PMID: 31514318 PMC6769434

[ref36] Zieba D. A. Szczesna M. Klocek-Gorka B. Molik E. Misztal T. Williams G. L.. (2008). Seasonal effects of central leptin infusion on melatonin and prolactin secretion and on SOCS-3 gene expression in ewes. J. Endocrinol. 198, 147–155. doi: 10.1677/joe-07-0602, PMID: 18451065

